# Thyroid radiation shields: A potential source of intraoperative infection

**DOI:** 10.1016/j.jor.2020.06.010

**Published:** 2020-06-19

**Authors:** T. McAleese, J.M. Broderick, E. Stanley, R. Curran

**Affiliations:** a St. James's Hospital, Dublin, Ireland; b Mater Misericordiae University Hospital, Dublin, Ireland; c Our Lady of Lourdes Hospital, Drogheda, Ireland

**Keywords:** Orthopaedic infection, Surgical site infection, Infection control, Thyroid radiation shields, Microbiology, Equipment contamination, Disinfection

## Abstract

**Introduction:**

Thyroid radiation shields are often uncovered by the surgical gown and may represent a preventable source of wound contamination. The aims of this study are to define the common pathogens found on thyroid radiation shields and evaluate the effectiveness of a simple cleaning method.

**Methods:**

Samples were obtained from 29 community thyroid shields collected from the operating theatres of 3 teaching hospitals. Each shield was swabbed under strict sterile technique using a separate swab for each of 4 designated zones. After sampling, shields were cleaned with a readily available disinfectant and sampling was repeated after 5 min. All swabs were cultured in ambient air at 37 °C on 5% sheeps blood agar for 48 hrs and subsequent growth was identified by a MALDI-TOF Walkaway mass spectrometer.

**Results:**

Before cleaning, 100% of thyroid shields (29/29) and 68% of shield zones (79/116) grew at least 1 type of bacteria. Coagulase negative staphylococci, including *S. epidermidis, S. capitis, S. cohnii, S. haemolyticus* and *S. hominis*, were most commonly isolated. *Enterobacteriaceae* and *S. aureus* were also cultured. After cleaning, culturable contamination was reduced by 86.3% and 64.5%, respectively (p < 0.001).

**Conclusion:**

The most common pathogens associated with SSIs can be isolated on thyroid radiation shields. Appropriate cleaning of thyroid shields with readily available disinfectant can significantly reduce the bacterial burden as detectable by culture. Hospitals should facilitate staff education and reinforce their policies on cleaning these shields which may often be overlooked.

## 1 Introduction

Despite modern infection control guidelines, surgical site infections (SSIs) remain the leading cause of nosocomial infection and represent a substantial burden of disease.^1^ In Europe, the percentage of SSIs per 100 surgical procedures can range from 0.6% to 9.5% depending on procedure type.^2^ Post-operative infections in orthopaedic patients can be particularly devastating given the setting of traumatized soft tissue and the frequent use of metal implants. Airborne transmission of bacteria is well recognised as a major contributor to SSIs.^3^^,^^4^ Exogenous bacteria originate from the shedding of exposed skin and hair of operating theatre personnel and contribute to infection by either direct contamination or by settling on inanimate objects before being introduced to the surgical wound.^5^, ^6^, ^7^ This has driven the implementation of numerous measures to prevent airborne bacterial load such as the use of laminar air-flow, hooded exhaust equipment, ultraviolet light cleaning and regular glove changing. Reducing the number of personnel in the operating theatre has also been shown to reduce the quantity of airborne bacteria.^8^

Lead garments are commonly worn in the operating theatre during orthopaedic procedures to facilitate the use of intraoperative fluoroscopy. At many institutions, these are provided by the hospital. They are stored on public hangers or shelves and are considered “shared-use”, increasing the risk of bacterial colonisation. These pathogenic bacteria can remain virulent for weeks to months on these surfaces. *Staphylococcus Aureus*, for instance, has been shown to remain infective for 10 days and some strains of multiresistant *S. aureus* (MRSA) have been shown to remain viable on cotton materials for up to 9 weeks.^9^^,^^10^

**Fig. 1:**
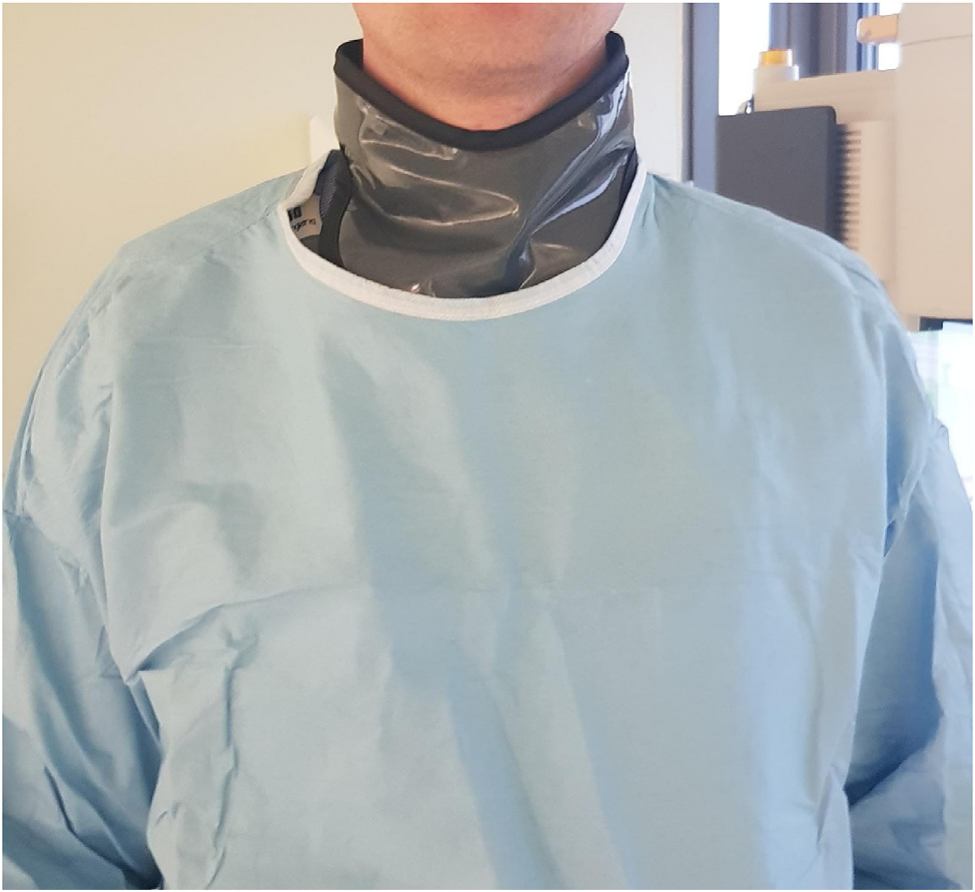
Thyroid radiation shields are often exposed to the sterile field above surgical gowns.

Thyroid radiation shields constitute an important part of radiation protection garments. However, potentially contaminated thyroid shields are often exposed above sterile surgical gowns (Fig. 1) and may represent a significant breach in the sterile field. Airborne transmission of bacteria harboured on thyroid radiation shields could increase the risk of intraoperative infection.

## 2 Objectives

We designed this study (1) to isolate and identify bacteria present on shared-use thyroid shields; (2) to determine the most contaminated region of the shields; and (3) to evaluate the effectiveness of a readily-available simple cleaning method.

## 3 Methods

This study was conducted across three university teaching hospitals, each of which is a Level II trauma centre. All thyroid shields were collected at the end of a routine weekday without informing any theatre staff to eliminate any bias due to the Hawthorne effect. This also ensured all shields were available at the time of sample collection.

**Fig. 2:**
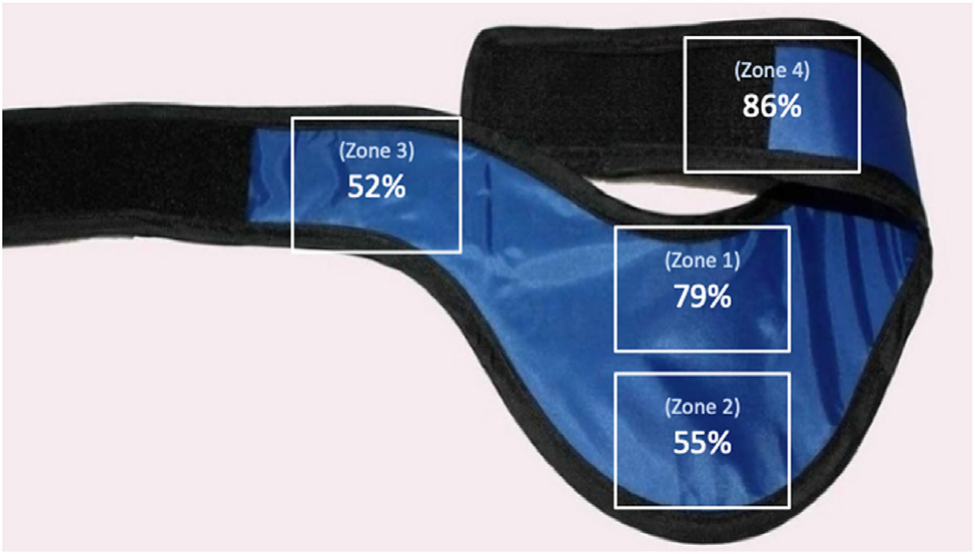
Percentage of thyroid shield contamination by quadrant.

The shields were brought into an operating theatre that had undergone a ‘deep clean’ and distributed on a sterile surface. The external surface of each shield was divided into four (2 × 2 cm) zones (Fig. 2): Zone 1 (Upper), Zone 2 (Lower), Zone 3 (Right Lateral) and Zone 4 (Left Lateral, including the velcro strap). Sterile gloves and a surgical mask were worn throughout sample collection and swabbing. We used media free, sterile swabs *(BBL™ CultureSwab™ EZ, Ireland)* and pre-moistened them in normal saline to facilitate extraction of any contaminant on the shield surface. Separate sterile swabs were used to sample each of the 4 zones.

As part of a well-accepted semi-quantitative technique, we used columbia blood agar supplemented with 5% sheep blood *(Columbia blood agar, Fannin Healthcare, Ireland).* This is a non-selective media known to support the growth of numerous bacterial strains, including Gram-negative and Gram-positive species. We inoculated each plate using the 4-quadrant streak method and labelled each plate appropriately. The thyroid shields were then cleaned with the hospital's medical-standard alcohol wipes *(Clinell universal disinfectant wipes, Gammahealthcare, UK)* and left for 5 min to dry ensuring full effect of the cleaning product. Each zone was then swabbed again using the previously described technique. Plates were incubated in ambient air at 37 °C and 5% CO2 for 48 h, after which they were examined for growth of bacterial colonies. All isolates were placed on a MALDI-TOF walkaway mass spectrometer for identification under the supervision of a consultant microbiologist.

A total of 232 swabs were taken (116 initial swabs and 116 post-disinfection swabs). The results were divided into *Staphlycoccus Aureus, Coagulase Negative Staphylococci (CoNS), Enterococcus species, Streptococcus viridans species, gram-positive bacilli, Fungal Species* and other bacteria. Positive growth was graded by the number of colony forming units (CFU) present. Standard 90 mm plastic petri dishes were categorised into: no growth, moderate growth 1+ (10–100 CFU), heavy growth 2+ (100–1000 CFU) and confluent growth 3+ (>1000 CFU).

### 3.1 Statistical analysis

We used the McNemar test to compare the contamination rates of the thyroid shields, as well as each of the 4 allocated zones, before and after decontamination. To determine if there was a significant difference in the contamination rate between zones before cleaning, we used the partitioned chi-squared independence test. Post-hoc correction analysis was performed using the Holm-Bonferroni method. Categorical data is presented in terms of number and percentage. Continuous data is categorised and presented in terms of number and percentage. We used SPSS 25.0 software *(SPSS, Chicago, IL)* for all analysis.

## 4 Results

29 thyroid radiation shields (116 zones) were swabbed and cultured before and after cleaning. Prior to decontamination, bacteria were isolated from 100% (29/29) of the thyroid radiation shields and 68% (79/116) of the shield zones After disinfection, bacteria were isolated from 13.7% (4/29) of the thyroid shields and this involved only one zone on each of these shields: 3.4% (4/116).

The post-disinfection reduction in both the number of contaminated thyroid shields (86.3%) and contaminated quadrants (64.6%) was statistically significant (p < 0.001).

The contamination rate between thyroid shield zones before cleaning was also statistically significant (x^2^ [3, N = 4] = 11.866, p < 0.008). Post-hoc analysis demonstrated that Zone 4 (left lateral including the velcro strap) was the most contaminated area with significantly more growth than Zone 2 (x^2^ = 6.74, p < 0.047) and Zone 3 (x^2^ = 8.06, p < 0.027). Bacteria was cultured on 86% (25/29) of swabs from Zone 4 and it was the most common to exhibit confluent growth (>1000 CFU). Zone 1, the area that sits just above the sterile gown, facing the operative field was also heavily contaminated and bacteria were present in this zone on 79% of the thyroid shields. The majority of contamination in Zone 1 cultured moderate or heavy growth of bacteria (Table 1).

**Table 1:** Contamination rates (%) of 29 thyroid shields divided by zone.

Pre-decontamination bacterial growth				
Quantity	Zone 1	Zone 2	Zone 3	Zone 4
1+	45%	31%	38%	48%
2+	31%	17%	14%	28%
3+	3%	7%	0%	10%
Total	79%	55%	52%	86%
Post-decontamination bacterial growth				
Quantity	Zone 1	Zone 2	Zone 3	Zone 4
1+	7%	0%	0%	3%
2+	0%	3%	0%	0%
3+	0%	0%	0%	0%
Total	7%	3%	0%	3%
**p value**	p < 0.001	p < 0.001	p < 0.001	p < 0.001

In 67% (53/79) of the contaminated zones, growth was polymicrobial, inoculating more than one pathogen. We classified bacterial and fungal growth by morphology and gram stain (Table 2). The majority of bacteria cultured on thyroid radiation shields are upper respiratory tract and skin commensals. The most common pathogens were coagulase negative staphylococci (CoNS) including *S. epidermidis, S. capitis, S. cohnii, S. hameolyticus* and *S. hominis.* CoNS were present on 29/29 (100%) of the shields and 57% (66/116) of quadrants before cleaning. Streptococcus viridans species including *Strep. mitis, Strep. oralis, Strep. parasangunis, Strep. sanguinis* and *Strep. infantis* were cultured on 9% of thyroid shield quadrants. Gram positive spore-forming bacilli including *Bacillus ceres, Bacillus lichenformis* and *Bacillus mycoides* were also isolated on 9% (10/116) of the zones. Staphylococcus Aureus was cultured on 3% of quadrants. Enterobacteriaceae and Candida Sp. were both cultured on 1 zone. Other respiratory pathogens such as *Micrococcus luteus*, *Rothia Sp.* as well as gram negative diplococci such as *Morexella catharhalis* and *Moraxella osteonsis* were also isolated.

**Table 2:**
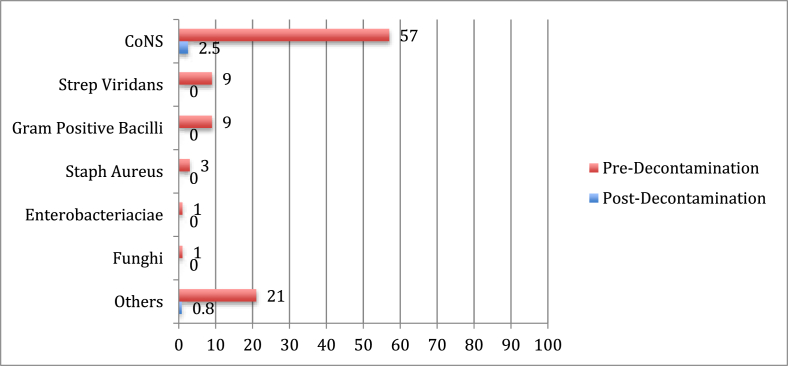
Percentage of quadrants contaminated on thyroid shield.

CoNS: Coagulase Negative Staphylococcus.

After decontamination with alcohol based solution, CoNS was present on 2.5% (3/116) of the quadrants as detected by the MOLDI-TOF mass spectrometer. CoNS were the most likely organisms to be present after disinfection, making up 75% of the residual bacteria present. The only other bacteria present after cleaning was Escherichia Coli.

## 5 Discussion

Reducing the rate of SSI is of paramount importance both from a societal and an economic perspective as they significantly increase healthcare cost, in-hospital length-of-stay, morbidity and mortality. In orthopaedic surgery, hospital acquired infection (HAI) increases patient length of stay by more than 2 weeks and increases costs by 300%.^11^ Furthermore, periprosthetic joint infection (PJI) has recently been demonstrated to have a five-year mortality of 21.12%.^12^ Patient factors, prophylactic antibiotics and length of stay are well documented contributors to the rate of healthcare associated infections. (7) However, recent literature demonstrates that intraoperative factors such as prolonged operation duration or repetitive minor breaches in sterile technique may have a larger impact on SSI rates than any patient factors.^7^ The extent of this risk and of that posed by healthcare workers acting as vectors, is less well known. It is clear, however, that equipment coming into direct contact with multiple healthcare providers and patients can be colonised with harmful bacteria^13^ and although operating personnel wear sterile gowns over protective lead garments, thyroid radiation shields are often exposed above these garments.

*Coagulase negative Staphylococci, Staphlycoccus Aureus* and *Streptococci* species are well recognised as the major pathogens associated with surgical site infection and periprosthetic joint infection.^14^, ^15^, ^16^ Our study demonstrated these were the most common pathogens isolated on thyroid radiation shields. As a result, we may be inadvertently placing a contaminated item above the operating field. A number of similar studies support our results. Feierabend et al. found that before cleaning, 81% of thyroid shields grew at least one type of bacteria and 90% of these were CoNS. Post-cleaning, contamination was reduced by 70%.^17^ Jaber et al. demonstrated that 100% of 25 lead garments and thyroid shields swabbed were contaminated with CoNS with 28% having more than 20 CFUs. They also isolated *Staphylococcus Aureus* on 84% of lead garments, 14% of which were methicillin resistant (MRSA).^18^ Furthermore, Ang et al. studied the contamination rates of lead garments in a high volume cardiac catheterisation laboratory and found that thyroid radiation shields harboured the most bacteria of any lead garments, the majority of which were CoNS.^19^ An abstract presented by Elsayed et al. demonstrated the presence of coagulase-negative *Staphylococci,* diptherioids, and aerobic spore-bearing organisms on 18 of 19 lead garments worn in the operating room. The authors reported that bacteria can survive on lead aprons and recommended stricter guidelines for decontamination between procedures.^20^ Interestingly, Grogan et al. found a very low rate of bacterial growth (2.7%) on sampled lead garments but could relate these findings to the organized and detailed disinfection protocols used in their institution.^13^

The most common CoNS isolated in our study were *Staphylococcus epidermidis* followed closely buy *Staphylococcus capitis.* The main virulence factors of these organisms are their ability to produce biofilms and surface adhesins, both facilitating antibiotic resistance. Biofilms function as an extracellular polysaccharide matrix barrier against host defences allowing the colonisation of biomaterials. Since the implementation of new identification methods such as matrix-assisted laser desorption/ionisation time-of-flight mass spectrometry (MALDI-TOF MS) or real time broad range polymerase chain reaction (PCR) focus has shifted to reporting CoNS to the species level and understanding the epidemiology and pathogenicity of each bacteria in PJI. *Staphlycoccus capitis* is a recognised cause of PJI requiring revision surgery and is thought to contracted during surgery or in the immediate post-operative period.^21^^,^^22^ It has also been shown to be the predominant CoNS in laminar air flow during prosthetic joint surgery.^23^

We also isolated a number of other potentially harmful strains from the shields, including *Enterococcus faecalis, Escherchia coli* and *Candida albicans.* Growth of Candida Sp. is particularly worrying as they are often non-susceptible to Cefuroxime and other commonly used prophylactic antibiotics for orthopaedic procedures. *Escherechia coli* is a member of the large family of gram negative bacteria encompassing enterobacteriaciae. An increasing number of hospital acquired infections are related to antibiotic-resistant pathogens such as carbopenum resistant enterobacteriaciae (CRE). This may reflect an increasing number of severely ill surgical patients or the widespread use of broad spectrum antibiotics and poor stewardship.^24^

We found that the velcro strap (Zone 4) and upper zone (Zone 1) were the most heavily contaminated areas on the thyroid shield. They were also the most likely area to contain any bacteria. We felt this correlated with the most frequently handled areas of the thyroid shield and the areas closest to the surgeon's skin. This is also in keeping with Bible et al.’s conclusion that the area just below the neck is the least sterile area of the surgical gown.^25^ They explained that this is due to transmission of bacteria from the face and neck onto this portion of the gown.

The main strengths of our study are the multi-centre sampling and using the Maldi-TOF mass spectrometer to allow for a more accurate identification and quantification of bacteria present. Our study was also well-designed, systematic and involved a good sample size. We took measures to reduce bias such as random sampling of thyroid shields and organising reporting of culture results by an independent scientist. We swabbed different areas of the thyroid shields including the heavily-handled velcro as these areas are often the most exposed. As a result, out study highlights a potential preventable source of surgical site infection and periprosthetic joint infection.

Limitations of the study include the fact we only measured one common method of disinfection. We did not define the antibiotic sensitivities or potential resistance of the some our cultures. We also sampled a range of thyroid shields with differing characteristics, from a number of manufacturers. We do not have baseline data regarding the ability of bacteria to colonise the different shield surfaces.

## 6 Conclusion

The results of this research suggests that a number of pathological strains thrive on thyroid radiation shields that are exposed to the surgical field. This may represent a preventable cause of post-operative infection. Continuous training and auditing of theatre staff around the prevention of infection is warranted. A specific sanitisation procedure for the thyroid radiation shields should be developed and adhered to by the cleaning staff in hospitals. Furthermore, a separate cabinet holding lead protective garments including thyroid radiation shields may reduce contamination and unnecessary handling.

## Funding

This research did not receive any specific grant from funding agencies in the public, commercial, or not-for-profit sectors.

## Declaration of competing interest

All of the authors named above in this publication have no conflicts of interest to declare.
